# Health-related quality of life profiles in patients with rheumatoid arthritis: a latent profile analysis

**DOI:** 10.3389/fpubh.2024.1478376

**Published:** 2024-12-18

**Authors:** Yuqing Song, Yanling Chen, Liting Wen, Benyi He, Yulin Ding, Mei Liu, Fangmei Tang, Li Wang, Jianmei Wu, Xue Deng, Lu Xing, Wen Zhao

**Affiliations:** ^1^Department of Gynecological Nursing, West China Second University Hospital, Sichuan University, Chengdu, Sichuan, China; ^2^Key Laboratory of Birth Defects and Related Diseases of Women and Children (Sichuan University), Ministry of Education, Chengdu, Sichuan, China; ^3^Department of Rheumatology and Immunology, West China Hospital, Sichuan University, Chengdu, Sichuan, China; ^4^Department of Neurosurgery, Shenzhen Second People’s Hospital, The First Affiliated Hospital of Shenzhen University, Shenzhen, China; ^5^Department of Hepatobiliary Pancreatic Vascular Surgery, The First Hospital of Kunming, Kunming, Yunnan, China; ^6^School of Nursing, Chengdu University of Traditional Chinese Medicine, Chengdu, China; ^7^Department of Rheumatology and Immunology, Deyang People’s Hospital, Deyang, Sichuan, China; ^8^Department of Obstetric Nursing, West China Second University Hospital, Chengdu, Sichuan, China; ^9^Department of Rheumatology and Immunology, Hospital of Chengdu University of Traditional Chinese Medicine, Chengdu, Sichuan, China

**Keywords:** rheumatoid arthritis, quality of life, latent profile analysis, physical function, disease activity

## Abstract

**Background:**

Rheumatoid arthritis (RA) is a common rheumatic disease that most commonly affects joints and negatively impacts individuals’ health-related quality of life (HRQoL). Although some studies have explored HRQoL of RA patients, existing studies treated RA patients as a homogeneous group based on their overall HRQoL and ignore the heterogeneity of patients’ HRQoL patterns. This study aimed to identify subgroups of RA patients based on their HRQoL and variables associated with group membership.

**Methods:**

This was a multi-center cross-sectional study conducted at 3 tertiary hospitals. All participants completed standardized questionnaires including demographic variables, HRQoL, physical function, disease activity and self-efficacy. Latent profile analysis was used to identify the optimal number of subgroups (profiles) and multinomial logistic regression analysis was used to explore variables associated with profile membership.

**Results:**

The analysis revealed 3 profiles of RA patients: poor HRQoL (*N* = 92, 60.9%), moderate HRQoL but poor role function (*N* = 45, 29.8%), good HRQoL (*N* = 14, 9.3%). Regression analysis revealed that patients with worse physical function were more likely to belong to “poor HRQoL” and “moderate HRQoL but poor role function” profile. Additionally, patients with junior high school or below educational level were less likely to belong to “moderate HRQoL but poor role function” profile.

**Conclusion:**

This study identified 3 profiles of HRQoL within RA patients and found that physical function and educational level was associated with HRQoL profiles. The finding can provide the basis for developing tailored interventions to specific subgroups of RA patients.

## 1 Introduction

Rheumatoid arthritis (RA) is one of the most common chronic autoimmune diseases (1). The prevalence of RA in the worldwide ranges between 0.5 and 1.0%, and approximately 5 million people in China suffer from this disease (1, 2). RA is characterized by joint pain, stiffness and swelling, leading to joint damage and disability, which limited patients’ ability to perform daily activities and work, such as writing, dressing, and walking (3). Previous studies revealed that RA patients are at a higher risk for developing psychological disorders (e.g., depression, anxiety) due to the long-term and unpredictable RA (4, 5). RA patients also occur sickness absence and work disability due to the symptoms of RA (6). Thus, RA significantly impacts the physical, psychological, and social aspects of patients’ daily lives, and negatively affects patients’ health-related quality of life (HRQoL). RA is uncurable, and the treatment goal of RA is to optimize HRQoL through controlling disease and minimizing the impact of the disease (7). HRQoL is a multidimensional concept that encompasses an individual’s perception of their position in life in the context of the culture and value systems in which they live, and in relation to their goals, expectations, standards and concerns (8). Incorporating HRQoL assessment into rheumatology care may help healthcare providers develop more patient-centered intervention and improve RA patients’ health-related outcomes (9).

Previous studies have explored the HRQoL and its associated factors in patients with RA (9, 10). These studies (10–13) revealed that RA patients’ HRQoL was more impaired compared with general population, spondyloarthritis patients, and physical component of HRQoL was more impaired than mental component. The Medical Outcomes Study 36-item Short-Form Health Survey (SF-36) was the most widely used tool to assess RA patients’ HRQoL. SF-36 includes 36 items, covering 8 domains: physical functioning (PF), role physical (RP), bodily pain (BP), general health (GH), vitality (VT), social functioning (SF), role emotional (RE), and mental health (MH) (14). The studies (12, 13) focusing on HRQoL in RA patients predominately evaluated the level of HRQoL and the demographic and clinical factors associated with HRQoL. These studies presented RA patients’ HRQoL as an overall score, which limited understanding of nuances in the diverse HRQoL and the utility of these scores as screen tool for health outcomes (15). Thus, exploring distinct patterns of HRQoL across multiple domains may help healthcare providers understand RA patients’ HRQoL well.

Latent profile analysis (LPA) is a personal-centered approach to identify subgroups within a population based on patterns of responses across multiple variables (16). LPA has been successfully used in diverse population, such as older patients (17), connective tissue diseases (CTD) (18), and students (19). Dyball et al. (18) evaluated the HRQoL profiles of CTD patients, and identified 3 latent profiles, including poor, average, and excellent HRQoL. This method enables us to identify whether there are distinct groups of individuals with similar patterns of HRQoL (17, 18). LPA can identify specific HRQoL profiles and who are most in need of intervention, which can provide basis for developing targeted interventions and enhance the effectiveness of intervention strategies.

To our knowledge, no published study used LPA specifically to identify HRQoL profiles in Chinese patients with RA. Identifying RA patients’ HRQoL profiles can contribute to developing targeted interventions to prompt HRQoL for RA patients. Based on these findings, this study aimed to use LPA to identify distinct HRQoL profiles in RA patients, and to explore demographic and clinical factors associated with different subgroups of HRQoL.

## 2 Methods

### 2.1 Study design

This study was a multicenter cross-sectional study conducted in three tertiary hospitals, including West China hospital, Deyang people’s hospital, and Hospital of Chengdu University of Traditional Chinese Medicine. This study recruited RA patients from November 2021 and March 2022.

### 2.2 Ethical considerations

This study was conducted in accordance with the Helsinki Declaration. Ethical approval was obtained from West China Hospital Medical Ethics Committee (ID: 20211368). This study also received permission from the other two hospitals. All participant provided written informed consent before they participated this study.

### 2.3 Participants

Participants were recruited from the Rheumatology and Immunology Departments of the three tertiary hospitals. We included participants if they: (1) were diagnosed with rheumatoid arthritis according to EULAR/ACR classification criteria (20); (2) were at least 18 years old; (3) could read or communicate in Chinese; (4) were willing to participate in this study. We excluded participants if they had: (1) other severe disease that may affecting HRQoL; (2) mental or cognitive impairment.

### 2.4 Measures

#### 2.4.1 Socio-demographic and disease-related characteristics

The socio-demographic and disease-related variables included age, gender, educational level, marital status, *per capita* monthly household income, symptom duration, diagnosis duration, and medication use. Medication mainly included conventional disease-modifying antirheumatic drugs (cDMARDs), biological or targeted synthetic DMARDs (b/tsDMARDs), and traditional Chinese medicine.

#### 2.4.2 Health-related quality of life

Health-related quality of life was assessed using the Chinese version of the Medical Outcomes Study Short Form 36-item Health Survey (SF-36) (21). This instrument can evaluate diverse aspects of individual’s well-being and health status. SF-36 includes 36 items with eight domains: physical functioning (PF), role physical (RP), bodily pain (BP), general health (GH), vitality (VT), social functioning (SF), role emotional (RE), and mental health (MH) (14). The 8 domains were summarized into two summary score: Physical Component Summary (PCS) and Mental Component Summary (MCS) (22). The scores of 8 domains and 2 component summaries ranges from 0 to 100, and higher scores indicate better HRQoL (14). SF-36 has been widely used to measure HRQoL of diverse population, such as general population, RA patients, and arthritis patients (21, 23, 24).

#### 2.4.3 Self-efficacy

Self-efficacy was measured by Chinese version of Arthritis Self-Efficacy Scale-8 (ASES-8) (25). ASES-8 was developed from the original 20-item ASES which includes 3 subscales related to pain, function, and other symptoms. The ASES-8 comprises 2 items from pain subscale, 4 items from other symptom subscale, and 2 new items related to preventing pain and fatigue from interfering with things the patients want to do (25, 26). Each item is scored from 1 (very uncertain) to 10 (very certain) based on patients’ ability to deal with symptoms of arthritis (27). The score of ASES-8 ranges from 1 to 10, and a higher score of ASES-8 represents higher self-efficacy (26). The ASES-8 had good reliability and validity in RA patients (25, 27).

#### 2.4.4 Disease activity

Disease activity was measured by Clinical Disease Activity Index (CDAI) (28–30). CDAI is a widely used tool for assessing disease activity in patients with RA. This index comprehensively considers 4 key indicators, including swollen joint count (SJC), tender joint count (TJC), patient’s global visual analog scale (PGV) and physician’s global visual analog scale (PhGV) (30). In the context of CDAI, disease states are defined by specific scoring ranges: remission (CDAI ≤2.8), low disease activity (2.8 < CDAI ≤10), moderate disease activity (10 < CDAI ≤22), and high disease activity (CDAI >22). CDAI is widely used in clinical trials and research (30).

#### 2.4.5 Physical function

Physical function was measured by health assessment questionnaire (HAQ) (31). HAQ is widely utilized to evaluate functional status in patients with arthritis (32, 33). HAQ includes 20 items to assess patients’ dressing and grooming, arising, eating, walking, hygiene, reaching, griping, and errands and chores (32). Each item is scored on a scale ranging from 0 to 3, where 0 represents no difficulty, and 3 indicates inability to perform the task. The 20 items are categorized into 8 functional categories with each category given a single score equal to the maximum value of their component items (33). The HAQ score ranges from 0 to 3, and a higher scores reflects worse physical function (34).

### 2.5 Data collection

We selected investigators worked in the selected hospitals to ensure patients’ privacy and quality of data collection. Investigators worked in the selected hospitals were fully informed about patient confidentiality requirements, which could ensure that the process of data collection adhered strictly to privacy regulations. Additionally, investigators in the hospitals have direct access to patients and medical records, which allowed the investigators to collect data accurately. Thus, we selected the investigators worked in the selected hospitals. Then, we trained the investigators about the research protocol and the content of questionnaires. Trained investigators collected data using the printed questionnaires and checked the completed questionnaires. Investigators invited patients to participate this study when RA patients come to hospital. Patients completed the questionnaires after they agreed to participate and provided informed consent form.

### 2.6 Statistical analysis

Data analyses were performed using SPSS Statistics Version 26.0 (IBM Corp., Armonk, NY, USA) and Mplus Version 7.0 (Muthén and Muthén, Los Angeles, CA, USA). Latent profile analysis (LPA) is a statistical method for identifying homogeneous subgroups of individuals based on a set of continuous measured variables. LPA was conducted to classify the participants into subgroup with respect to the eight domains of SF-36. A single profile was initially executed, and this profile number gradually increased to 4. We used the Bayesian information criterion (BIC), the Akaike information criterion (AIC), and the sample size adjusted Bayesian information criterion (SABIC), bootstrap sequential likelihood ratio test (BLRT), and Vuong-Lo-Mendel-Rubin adjusted likelihood ratio test (LMRT) to compare fits of models and numbers of latent profiles (LP). Entropy was reported to evaluate the classification accuracy, and values ≥0.8 indicated a good profile solution. A one-way analysis of variance (ANOVA), Kruskal-Wallis and *χ*^2^ test was conducted to determine whether there were differences in variables across the profiles. Multinomial logistic regression analysis was used to explore the relationships between patient-level characteristics and LP membership, with profile 3 (good HRQoL) as the reference group.

## 3 Results

### 3.1 Participants’ characteristics

We included 151 RA patients with a mean age of 57.97 (SD = 11.64, ranging from 20 to 86) years. Most of the participants were female (72.8%), married (86.8%). Around half participants had primary school or below (45.0%) educational level and income < ￥2000 (58.3%). The median symptom and diagnosis duration were 6, 4 years, respectively. The mean scores of disease activity and self-efficacy were 22.32 and 4.75, respectively. We also described 8 domain score of SF-36. Participants’ characteristics are presented in Table 1.

**Table 1:** Participant characteristics (*N* = 151).

Variables	Mean ± SD	Range
Age	57.97 ± 11.64	20–86
Variables	*N* (%)	
Gender		
Male	41 (27.2%)	
Female	110 (72.8%)	
Marital status		
Married	131 (86.8%)	
Single/Divorced/Widowed	21 (13.2%)	
Educational level		
Primary school or below	68 (45.0%)	
Junior high school	51 (33.8%)	
Senior high school or above	32 (21.2%)	
Income (￥)		
< 2000	88 (58.3%)	
2000 ~ 4,000	48 (31.8%)	
> 4,000	15 (9.9%)	
Medication use		
cDMARDs (yes)	80 (53.0%)	
b/tsDMARDs (yes)	33 (21.9%)	
Traditional Chinese medicine (yes)	100 (66.2%)	
Variables	Mean ± SD/Median (IQR)	Range
Symptom duration (years), median (IQR)	6.00 (15.00)	0 ~ 51
Diagnosis duration (years), median (IQR)	4.00 (13.50)	0 ~ 47
Disease activity, mean ± SD	22.32 ± 10.97	
Physical function, median (IQR)	0.75 (1.63)	0 ~ 3
Self-efficacy, mean ± SD	4.75 ± 2.04	0 ~ 69
Quality of life		
PF, mean ± SD	51.45 ± 29.23	0 ~ 100
RP, median (IQR)	0 (0)	0 ~ 100
RE, median (IQR)	0 (33.30)	0 ~ 100
VT, mean ± SD	58.01 ± 21.39	5 ~ 100
MH, mean ± SD	58.07 ± 22.09	12 ~ 100
SF, mean ± SD	55.25 ± 31.75	0 ~ 100
BP, mean ± SD	31.52 ± 19.63	0 ~ 90
GH, mean ± SD	38.51 ± 19.23	0 ~ 90

SD, standard deviation; IQR, interquartile range; HRQoL, health-related quality of life; PF, physical functioning; RP, role-physical; BP, bodily pain; GH, general health; VT, vitality; SF, social functioning; RE, role-emotional; MH, mental health; cDMARDs, conventional disease-modifying antirheumatic drugs; b/tsDMARDs, biological or targeted synthetic disease-modifying antirheumatic drugs.

### 3.2 The results of latent profile analysis

LPA were performed with the 8 domains of SF-36 as input variables. We tested the models with 1 to 4 profiles. The model fit statistics of the four LPA models are shown in Table 2. AIC, BIC, SABIC and entropy decreased with the increasing numbers of latent profiles. The entropy value was high (>0.8) for all latent profile models. The LMRT was statistically significant (*p* < 0.05) with 3-profile model. The BLRT values remained significant (*p* < 0.05) for all profile models. Based on these statistics, a 3-profile model was selected.

**Table 2:** Comparison of fit indices between models.

Model	AIC	BIC	SABIC	Entropy	LMRT	BLRT	Class counts
1	11250.103	11298.379	11247.741				151
2	10896.059	10971.491	10892.369	0.921	0.3697	0.0000	108/43
**3**	**10753.468**	**10856.056**	**10748.450**	**0.919**	**0.0428**	**0.0000**	**92/45/14**
4	10674.554	10804.297	10668.206	0.899	0.5057	0.0000	59/12/65/15

AIC, Akaike information criterion; BIC, Bayesian information criterion; SABIC, sample size-adjusted Bayesian information criterion; LMRT, Lo–Mendell–Rubin likelihood ratio test; BLRT, bootstrap likelihood ratio test. Bold indicates 3-profile model was selected.

**Figure 1:**
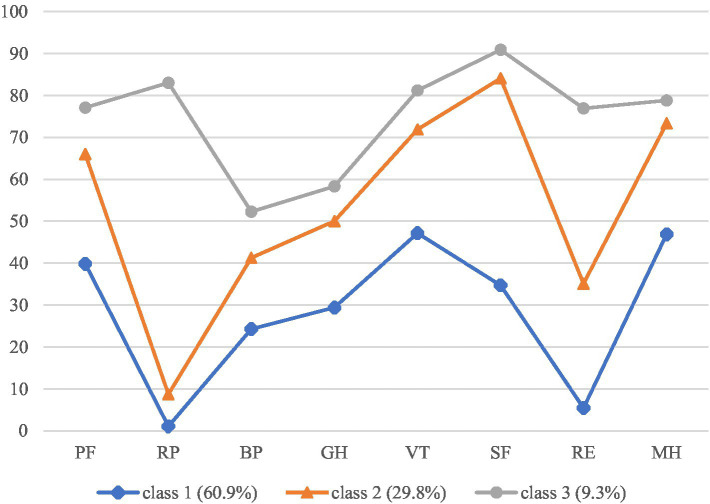
The three-profile mode of HRQoL. PF, physical functioning; RP, role-physical; BP, bodily pain; GH, general health; VT, vitality; SF, social functioning; RE, role-emotional; MH, mental health.

Table 3 and Figure 1 show the SF-36 domain scores of the 3-profile model. One-way ANOVA analysis and Kruskal-Wallis test revealed that there were significant differences between the 3 profiles for PF (*F* = 26.416, *p* < 0.001), RP (*H* = 86.748, *p* < 0.001), RE (*H* = 53.636, *p* < 0.001), VT (*F* = 46.400, *p* < 0.001), MH (*F* = 43.558, *p* < 0.001), SF (*F* = 137.911, *p* < 0.001), BP (*F* = 22.078, *p* < 0.001), GH (*F* = 41.987, *p* < 0.001). Profile 1 (*N* = 92, 60.9%) was characterized by low scores of all domains and was labeled “poor HRQoL.” Profile 2 (*N* = 45, 29.8%) was characterized by moderate scores of PF, BP, GH, VT, SF, MH, but poor RP and RE scores. Thus, profile 2 was labeled “moderate HRQoL but poor role function.” Profile 3 (*N* = 14, 9.3%) was characterized by high scores across all domains and was labeled “good HRQoL.”

**Table 3:** Comparison of socio-demographic and clinical variables among different health-related quality of life profiles.

Variables	Class 1	Class 2	Class 3	F/*χ*^2^/H	*p*
Age, mean ± SD	59.59 ± 11.75	56.93 ± 11.55	50.71 ± 8.19	3.935^a^	**0.022**
Gender, *N* (%)				1.298^b^	0.523
Male	26 (28.3%)	13 (28.9%)	2 (14.3%)		
Female	66 (71.7%)	32 (71.1%)	12 (85.7%)		
Marital status, *N* (%)				1.946^b^	0.378
Married	77 (83.7%)	41 (91.1%)	13 (92.9%)		
Single/Divorced/Widowed	15 (16.3%)	4 (8.9%)	1 (7.1%)		
Educational level, *N* (%)				10.114^b^	**0.039**
Primary school or below	50 (54.3%)	13 (28.9%)	5 (35.7%)		
Junior high school	26 (28.3%)	18 (40.0%)	7 (50.0%)		
Senior high school or above	16 (17.4%)	14 (31.1%)	2 (14.3%)		
Income, *N* (%)				5.755^b^	0.218
< 2000	59 (64.1%)	23 (51.1%)	6 (42.9%)		
2000 ~ 4,000	23 (25.0%)	19 (42.2%)	6 (42.9%)		
> 4,000	10 (10.9%)	3 (6.7%)	2 (14.3%)		
Medication use, *N* (%)					
cDMARDs (yes)	47 (51.1%)	24 (53.3%)	9 (64.3%)	0.853^b^	0.653
b/tsDMARDs (yes)	19 (20.7%)	11 (24.4%)	3 (21.4%)	0.252^b^	0.881
Traditional Chinese medicine (yes)	63 (68.5%)	30 (66.7%)	7 (50.0%)	1.773^b^	0.412
Symptom duration (years), median (IQR)	6.00 (15.25)	7.00 (12.50)	9.50 (14.00)	0.444^c^	0.801
Diagnosis duration (years), median (IQR)	4.00 (13.50)	3.00 (13.00)	9.00 (16.50)	0.675^c^	0.714
Disease activity, mean ± SD	23.94 ± 10.75	21.20 ± 11.29	15.29 ± 8.43	4.300^a^	**0.015**
Physical function, median (IQR)	1.38 (1.63)	0.38 (0.63)	0 (0.28)	45.633^c^	**<0.001**
Self-efficacy, mean ± SD	4.34 ± 1.82	5.25 ± 2.18	5.77 ± 2.35	5.188^a^	**0.007**
HRQoL					
PF, mean ± SD	39.72 ± 26.49	67.11 ± 23.66	78.21 ± 21.36	26.416^a^	**<0.001**
RP, median (IQR)	0 (0)	0 (25)	100 (31.25)	86.748^c^	**<0.001**
RE, median (IQR)	0 (0)	33.30 (66.70)	100 (41.65)	53.636^c^	**<0.001**
VT, mean ± SD	47.61 ± 18.09	72.00 ± 14.59	81.43 ± 15.25	46.400^a^	**<0.001**
MH, mean ± SD	47.40 ± 17.27	73.42 ± 16.58	78.86 ± 22.92	43.558^a^	**<0.001**
SF, mean ± SD	34.84 ± 20.04	85.83 ± 15.90	91.07 ± 19.87	137.911^a^	**<0.001**
BP, mean ± SD	24.57 ± 17.48	39.22 ± 16.06	52.50 ± 20.33	22.078^a^	**<0.001**
GH, mean ± SD	29.40 ± 14.00	51.00 ± 17.04	58.21 ± 19.18	41.987^a^	**<0.001**

HRQoL, health-related quality of life; PF, physical functioning; RP, role-physical; BP, bodily pain; GH, general health; VT, vitality; SF, social functioning; RE, role-emotional; MH, mental health. Bold indicates *p* < 0.05.

a one-way analysis of variance (ANOVA).

b *χ2 test.*

c Kruskal-Wallis test.

### 3.3 Factors associated with latent profiles

Table 3 shows that there were differences between profiles on age, educational level, disease activity, physical function, and self-efficacy (all *p* < 0.05). Table 4 outlines the results of multinomial logistic regression analysis identifying the association between participants’ characteristics and profiles. Patients with higher HAQ scores were more likely to classify into profile 1 (poor HRQoL; OR 61.162, 95%CI 4.066, 919.918) and 2 (moderate HRQoL but poor role function; OR 16.574, 95%CI 1.100, 249.725) compared with profile 3 (good HRQoL). RA patients with educational level of junior high school (OR 0.084, 95%CI 0.009, 0.762) and primary school or below (OR 0.118, 95%CI 0.014, 0.965) were less likely to belong to profile 2 (moderate HRQoL but poor role function) compared with higher educational level.

**Table 4:** Multinomial logistic regression analysis of factors associated with profiles.

Variables	Profiles 1		Profiles 2	
	OR (95% CI)	*p*	OR (95% CI)	*p*
Age	1.056 (0.984, 1.134)	0.133	1.070 (0.997, 1.148)	0.062
Self-efficacy	0.851 (0.607, 1.193)	0.350	0.996 (0.716, 1.385)	0.982
Physical function	61.162 (4.066, 919.918)	**0.003**	16.574 (1.100, 249.725)	**0.042**
Disease activity	1.069 (0.977, 1.170)	0.146	1.088 (0.995, 1.190)	0.063
Educational level (reference: Senior high school or above)				
Primary school or below	0.233 (0.025, 2.186)	0.202	0.084 (0.009, 0.762)	**0.028**
Junior high school	0.171 (0.020, 1.485)	0.109	0.118 (0.014, 0.965)	**0.046**

Reference group: profile 3. Bold indicates *p* < 0.05.

## 4 Discussion

This study aimed to use a patient-centered approach-LPA to identify HRQoL latent profiles in RA patients and determine patient-level characteristics (e.g., demographic and clinical factors) associated with profile membership. In the current study, LPA identified three distinct HRQoL profiles (poor HRQoL, moderate HRQoL but poor role function, good HRQoL). This result was consistent with previous studies (17, 18, 35). Băjenaru et al. (17) applied LPA to identify 3 distinct QOL profiles in older patients: low and very low, moderate, and high quality of life. Liu et al. (36) recruited 354 older adults from nursing home and identified 3 latent quality of life profiles: low quality of life with poor psychological health, moderate quality of life, and high quality of life. Dyball et al. (18) used SF-36 to detect 3 latent HRQoL profiles among patients with CTD. We also found that worse physical function and lower educational level were associated with poor or moderate HRQoL profiles. These findings could help healthcare providers develop more targeted interventions for patients with distinct HRQoL profiles.

The largest group (poor HRQoL) was characterized by low scores across all 8 domains of SF-36 and accounted for 60.9% of RA patients. The second largest group was “moderate HRQoL but poor role function,” accounting for 29.8% of RA patients. This profile was characterized by moderate scores in most domains but poor scores in RP and RE, which means patients in this group facing challenges related to role limitations due to physical and emotional health. The third profile was good HRQoL with high scores across all domains, accounting for 9.3% of RA patients. Our study found that the majority of RA patients had poor HRQoL. Previous studies (17, 36) revealed that the majority of older people had moderate HRQoL. Dyball et al. (18) found that 61.4% CTD patients reported average HRQoL. Our results confirmed that RA patients’ HRQoL were more impaired than general population and patients with other disease (11, 13, 37). Healthcare providers should provide more intensive care for patient classified into poor HRQoL profile, and recognize the unique needs of individuals with moderate HRQoL. Thus, tailored interventions should be conducted for patients classified into different latent profiles.

We also identified factors associated with latent profiles. In the current study, RA patients with worse physical function were more like to belong to poor and moderate HRQoL profile. This finding is in line with previous studies which reveals that worse physical function is associated with lower HRQoL (12, 38). Santos et al. (39) revealed that physical function is one of the major HRQoL determinants in spondyloarthritis patients. Carvalho et al. (38) revealed that physical function is the main contributor to HRQoL in patients with RA and spondyloarthritis (SpA). Pharmacological treatment, exercise interventions, and educational interventions are effective to improve RA patients’ physical function (40–44). Thus, complex interventions that incorporate multiple intervention component are required to prompt RA patients’ physical function and HRQoL. Our study also found that RA patients with the educational level of junior high school or below were less likely to belong to profile 2 compared with high educational level. Rao et al. (45) found that low sociodemographic status was positively associated with poor HRQoL among arthritis patients. The finding suggests that developing and conducting interventions for improving HRQoL should consider patients’ educational level.

### 4.1 Limitation

The strengths of this study are conducting multicenter study and focusing on a person-centered approach by using LPA to identify profiles of HRQoL, but there were also several limitations. Firstly, the cross-sectional study design cannot examine causation or examine temporal changes in HRQoL profiles. Longitudinal studies can be conducted to identify the predictors of the HRQoL profiles and how the profiles evolve over time. Secondly, the generalizability of this study may be limited by small sample size because all participants were recruited from three hospital of Deyang and Chengdu. Future multicenter studies with large sample size would enhance the external validity of the findings.

## 5 Conclusion

This study identified 3 distinct profiles of HRQoL among RA patients using LPA: poor HRQoL, moderate HRQoL but poor role function, good HRQoL. The majority of RA patients belonged to poor HRQoL. We found that educational level and physical function were associated with HRQoL profiles. Tailoring interventions based on the identified profiles can enhance the effectiveness of care, addressing the specific needs of each subgroup.

## Data Availability

The original contributions presented in the study are included in the article/supplementary material, further inquiries can be directed to the corresponding authors.
